# Protection against Autoimmune Diabetes by Silkworm-Produced GFP-Tagged CTB-Insulin Fusion Protein

**DOI:** 10.1155/2011/831704

**Published:** 2011-06-06

**Authors:** Qiaohong Meng, Wenfeng Wang, Xiaowen Shi, Yongfeng Jin, Yaozhou Zhang

**Affiliations:** ^1^Institute of Biochemistry, College of Life Sciences, Zhejiang University (Zijingang Campus), 388 Yuhangtang Road, Hangzhou 310058, China; ^2^Institute of Biochemistry, College of Life Sciences, Zhejiang Sci-Tech University, Second Avenue, Hangzhou 310018, China

## Abstract

In animals, oral administration of the cholera toxin B (CTB) subunit conjugated to the autoantigen insulin enhances the specific immune-unresponsive state. This is called oral tolerance and is capable of suppressing autoimmune type 1 diabetes (T1D). However, the process by which the CTB-insulin (CTB-INS) protein works as a therapy for T1D *in vivo* remains unclear. Here, we successfully expressed a green fluorescent protein- (GFP-) tagged CTB-Ins (CTB-Ins-GFP) fusion protein in silkworms in a pentameric form that retained the native ability to activate the mechanism. Oral administration of the CTB-Ins-GFP protein induced special tolerance, delayed the development of diabetic symptoms, and suppressed T1D onset in nonobese diabetic (NOD) mice. Moreover, it increased the numbers of CD4^+^CD25^+^Foxp3^+^ T regulatory (Treg) cells in peripheral lymph tissues and affected the biological activity of spleen cells. This study demonstrated that the CTB-Ins-GFP protein produced in silkworms acted as an oral protein vaccine, inducing immunological tolerance involving CD4^+^CD25^+^Foxp3^+^ Treg cells in treating T1D.

## 1. Introduction

Oral tolerance refers to the physiological response of an organism remaining in a state of specific immunological unresponsiveness to some food antigens that is orally administered [1]. Data indicate that this can be used to treat autoimmune diseases, like type 1 diabetes (T1D), which has specific postulated autoantigens, such as insulin and glutamic acid decarboxylase 65 [2–5]. Indeed, in animal models, oral administration of tissue-specific antigen insulin was able to prevent T1D [6]. However, a major problem remains to be solved if oral tolerance is employed to treat human autoimmune diseases: how to obtain sufficient amounts of autoantigens for repeated oral administration because humans have an enormous intestinal absorptive surface area. Thus, the effective use of oral tolerance to treat autoimmune diseases may depend on the use of mucosal adjuvants to enhance efficacy.

As a mucosal carrier, the nontoxic cholera toxin B subunit (CTB) is conjugated to autoantigens for the induction of oral tolerance [7]. It has also been demonstrated in similar systems [8–10] that conjugating antigen to CTB can increase efficiency and thus reduce effective antigen doses. The therapeutic applications of CTB-mediated oral tolerance, as demonstrated in animal models, include the prevention and treatment of T-cell-mediated autoimmune diabetes [11].

The intestinal mucosa is the central location for the induction of oral tolerance [12]. Orally administrated, cholera toxin (CT), a potent mucosal modulator, induces the appearance of cells that share some common traits with Peyer's patch (PP) M-cells in mice [13]. Glycoprotein 2 on M-cells serves as a transcytotic receptor for mucosal antigens [14]. Additionally, it has been shown that dendritic cells (DCs) in the gut-associated lymphoid tissue (GALT) take up antigen and transfer it to T cells to generate Treg cells in oral tolerance-treated arthritic mice [15]. Sublingual administration of CTB-conjugated antigen to B-cell-deficient mice sharply reduced CD4^+^CD25^+^Foxp3^+^ Treg cells compared with the wild type [16, 17]. Together, these findings suggest that the regulatory T cells elicited by mucosal immunization with CTB conjugated peptides are unique and distinct from those that arise from spontaneous endogenous priming. This protein vaccine therapy may be useful in treating autoimmune diseases.

T1D is a spontaneous autoimmune disease associated with the pancreas in which damage to the insulin-producing *β*-cells disturbs glucose metabolism and leads to a series of complications [18]. The autoantigen insulin conjugated to CTB produced in silkworms can induce oral tolerance, protecting against T1D in NOD mice [19]. Using the silkworm as a “bioreactor” to obtain large amounts of proteins through the insect baculovirus expression system (BES) has many merits [20, 21]. As the green fluorescent protein (GFP) is widely used to reveal intestinal epithelial immunoreaction by tagging antibodies or microorganisms [13, 14], we expressed a GFP-tagged CTB-Ins (CTB-Ins-GFP) protein by a Bac-to-Bac silkworm BES in this study. We found that the CTB-Ins-GFP fusion protein was efficiently produced in both BmN cells and silkworms and possessed the functional characteristics of native CTB. Oral administration of the protein was capable of inducing specific immune tolerance, delaying diabetes symptoms, and suppressing T1D onset in NOD mice. The specific tolerance could increase the Foxp3^+^ regulatory T cell proportions in peripheral lymph tissues and suppress the biological functions of spleen lymphocytes in mice. This silkworm-produced active CTB-Ins-GFP protein, administered as a vaccine protein, was able to induce insulin specific oral tolerance, which is related to increased Treg cells in the treatment of T1D. 

## 2. Material and Methods

### 2.1. Reagents, Cell Lines, Silkworms, and Mice

DNA manipulation and PCR amplification kits were purchased from TaKaRa Biomedicals (Japan). The rabbit anticholera toxin primary serum, bacterial CTB peptides, and monosialoganglioside-GM1 were from Sigma-Aldrich (USA). A mouse regulatory T-cell staining kit was obtained from eBioscience (USA). Transwell chambers (*∅* = 8 *μ*m) were purchased from Corning Incorporated (USA). The silkworm BmN cells were cultured in TC-100 medium (Gibco-BRL, USA) containing 10% fetal calf serum (Gibco-BRL, USA) and 50 *μ*g/mL gentamycin at 27°C. Fifth-instar silkworm* B. mori* larvae (Jingsong × Haoyue, Showa) were fed fresh mulberry leaves and reared under a photoperiod schedule of 12-h light and 12-h darkness at 25 ± 1°C. Female nonobese diabetic (NOD) mice and NOD severe combined immunodeficient (NOD/SCID) mice were purchased from Shanghai Laboratory Animal Center, Chinese Academy of Sciences (SLAC, CAS, China), and housed at the central animal facility, where they were screened for bacterial and viral pathogens. 

### 2.2. Construction of Recombinant Bacmid Vectors

Five primers were designed prior to the construction of the CTB-GFP and CTB-Ins-GFP fusion genes (Table 1). Using the recombinant PAK-CTB-INS plasmid and the pEGFP plasmid [22] as templates, the CTB-GFP and CTB-Ins-GFP fusion genes were amplified by splice overlap extension PCR (SOE-PCR), identified, and subcloned into the donor pFastBac1 plasmid. Then, the recombinant bacmid vectors were constructed and verified by PCR identification and fragment sequencing. 

### 2.3. Acquisition of Recombinant Baculovirus in BmN Cells

A subconfluent monolayer of BmN cells was transfected with the recombinant bacmid vectors using Lipofectamine 2000 (Invitrogen, USA). The recombinant virus was generated in the transfected BmN cells after 3–5 days and verified by detection of green fluorescent light under a fluorescent microscope (Nikon, Japan). Viral genomic DNA was then extracted using the Wizard genomic DNA purification kit (Promega, USA) and identified by PCR amplification and fragment sequencing. Prior to the next viral infection for fusion protein expression, the dilution of the recombinant virus was calculated using the Reed-Muench method. 

### 2.4. Expression and Collection of the Fusion Proteins

BmN cells (4 × 10^6^) were infected by BmNPV CTB-GFP and BmNPV CTB-Ins-GFP at MOI = 10 and collected at 2–7 days after infection. Harvested BmN cells were suspended in 0.5 mL phosphate-buffered Saline (PBS) and lysed by gentle sonication several times on ice. They were centrifuged and the supernatant removed and stored at −20°C. The fifth-instar silkworm larvae were needle inoculated with the viral solutions (1 × 10^7^ pfu/mL) into their body cavities. The hemolymph of the larvae were collected at 2–7 days after inoculation, centrifuged to remove insoluble impurities, and stored at −20°C. 

### 2.5. Western Blot and ELISA Assay

To detect the expression of monomeric or pentameric fusion proteins, the cell-lysed supernatants or hemolymph samples were diluted and separated by 12% SDS-PAGE. Samples were either boiled or loaded directly on the gel. The separated protein bands were then transferred to a nitrocellulose (NC) filter membrane. Detection of the immunoreaction was performed with an enhanced chemiluminescence (ECL) Western blotting kit (Biologicacl Industries, Israel). Rabbit anticholera toxin antiserum (Sigma, USA) and rabbit anti-GFP primary antibody (Epitomics, USA) were used for the immunoreactions.

A semiquantitative ELISA was used to investigate the expression level of the fusion protein. A 96-well microtiter plate was loaded with dilutions of the cell-lysed supernatant (1 : 20–1 : 100) or hemolymph (1 : 500–1 : 2000) in bicarbonate buffer, pH 9.6 (15 mM Na_2_CO_3_, 35 mM NaHCO_3_) at 4°C overnight. The plate was blocked with 1% BSA at 37°C for 1 h and then washed with PBS containing 0.05% Tween-20 (PBST). A 1 : 5000 dilution of anticholera toxin antiserum in 1% BSA was added at 37°C for 2 h. It was then washed with PBST and incubated with a 1 : 10000 dilution of antirabbit IgG conjugated with horseradish peroxidase at 37°C for 40 min. Finally, the chromogenic substrate O-phenylenediamine was added at 37°C for 30 min to develop color and 2 M H_2_SO_4_ (50 *μ*L/well) was added to stop the reaction. The absorbance at 492 nm was measured in a Labsystems Multiscan MS ELISA plate reader (Labsystems, Finland). Serial dilutions of bacterial CTB (Sigma, USA) were used to generate the standard curve to calculate the results. 

### 2.6. GM1 Ganglioside-Binding Assay

A GM1-ELISA was performed to detect the affinity of silkworm-derived fusion proteins for GM1 ganglioside. The microtiter plates were coated with monosialoganglioside-GM1 (Sigma, USA) by incubating the plates with 50 *μ*L/well of GM1 in methanol at 4°C overnight. The wells were then blocked with BSA solution, and the dilutions of hemolymph were added. The remainder of the procedure was identical to the semiquantitative ELISA assay described above. The same dilutions of normal hemolymph and serial dilutions of bacterial CTB were used as a negative control and to generate the standard curve, respectively. 

### 2.7. Induction of Oral Tolerance

Five-week-old female NOD mice were divided into four groups and fed with Saline, hemolymph synthesized CTB-GFP protein, CTB-Ins-GFP protein, or CTB-INS protein. Beginning at 5 weeks of age, the mice were fed an equal amount of hemolymph (about 100–300 *μ*L) every other day until 10 weeks of age. Each feeding of hemolymph delivered approximately 50 *μ*g of the corresponding fusion proteins. The animals were sacrificed at 10 weeks of age for antibody titer assays and pancreatic islet histopathological analysis. 

### 2.8. Pancreatic Islet Histopathological Analysis

To evaluate insulitis in experimental NOD mice, the extent of lymphocyte infiltration in the islets was measured. At 10 weeks of age, six mice in each group were sacrificed and the pancreas removed. Each pancreas was fixed in PBS-buffered paraformaldehyde, embedded in paraffin, cut into 5 *μ*m sections, and stained with hematoxylin and eosin. The degree of insulitis was evaluated using a standardized scoring system with two independent observers using a semiquantitative scale ranging from 0 to 4: 0, normal islet with no sign of T-cell infiltration, 1, focal peri-islet T-cell infiltration, 2, more extensive peri-islet infiltration but with lymphocytes less than one-third of the islet area, 3, intraislet T-cell infiltration in one-third to one-half of the islet area, and 4, extensive intraislet inflammation involving more than half of the islet area. At least 20 islets were scored for each animal. 

### 2.9. Assessment of Diabetes

Incidences of diabetes were compared among mice fed the CTB-INS, CTB-Ins-GFP, or CTB-GFP fusion proteins or Saline. The feeding schedule was the same as mentioned above and continued for 30 weeks. Starting at 10 weeks of age, the development of diabetes was monitored and confirmed by measuring blood glucose using the ACCU-CHEK III system (Roche Diagnostics Ltd., Shanghai, China). A mouse with a blood glucose level above 16.7 mM for 2 consecutive weeks was considered diabetic. 

### 2.10. Gut Mucosal Binding Assay

Mice were anesthetized with ether and kept warm on a 37°C warming pad during the assay. Silkworm-derived CTB-GFP or CTB-Ins-GFP fusion proteins were injected into the ligated intestinal loops. After incubation for 30–60 min, the mice were sacrificed and part of the intestinal loops (about 5 mm) was excised [14]. After washing, 8 *μ*m frozen sections of the specimens were prepared. The locations of the proteins were verified by fluorescent microscopy. 

### 2.11. Quantification of Serum Antibody Subtypes

Treated NOD mouse serum was quantified for anti-CTB and anti-insulin antibody subtype using ELISA. Human insulin (Novo Nordisk) or bacterial CTB were used as the well-coating antigens and after blocking, serial dilutions of pooled sera were added to the coated microtiter plate wells. Horseradish peroxidase-conjugated antimouse IgG1, IgG2a, IgA, or IgE antibody (SABC, China) was used as the secondary antibody. The absorption value was measured as described above. The titer was defined as the reciprocal of the highest dilution of the sample giving an absorption signal above background, and was individually determined for each sample. 

### 2.12. Cytometric Analysis of Treg Cell Flow

Lymphocytes in the spleen, intestinal lymph nodes, and blood of 10-week-old treated NOD mice were obtained with the help of mouse lymphocyte separation medium (Dakewe Biotech Company, Limited, China). Then, the assay was performed with a mouse regulatory T-cell staining kit, which contains FITC-antiCD4 antibody, PE-antiCD25 antibody, and PE-cy5-antiFoxp3 antibody, according to the manufacturer′s protocol. Proportions of Treg cells in the lymph tissues were analyzed by flow cytometric analysis of lymphocyte phenotypic markers with a FACScan flow cytometer (Beckman Coulter, USA). 

### 2.13. Spleen Lymphocyte BrdU Label Analysis

Spleen lymphocytes were cultured with 10 *μ*M 5-bromo-2-deoxyuridine for 1 day to label the proliferated cells as in a previous study [23]. They were then fixed and treated with Triton X-100 and H_2_O_2_, denatured with HCl, and neutralized with boric acid, all at room temperature. Following blocking, the cells were treated with mouse anti-BrdU antibody (Boster, China) then incubated with biotin-labeled antimouse IgG antibody. Next, ABC compound was added to the cells. DAB was used to develop the corresponding brown color with hematoxylin used as a counterstain. Samples were analyzed using a microscope at 400x. 

### 2.14. Spleen Lymphocyte Transwell Assay

Spleen lymphocytes (1 × 10^5^) were isolated from NOD mice, and cultured in the upper chamber of 24-well Transwell plates with RPMI 1640 serum-free medium. IL2 was added in the normal culture medium (150 IU/mL) and placed in the bottom chamber and the cells were cultured for about 14 h at 37°C with 5% CO_2_. Next, the cells were fixed and stained with crystal violet solution after the cells upon the chamber were clear. Samples were analyzed using a microscope at 400x. 

### 2.15. Adoptive Transfer of Diabetes

Splenocytes from 10-week-old NOD mice fed CTB-Ins-GFP fusion proteins were assessed for the capacity to suppress the diabetogenic activity of splenocytes taken from an acutely diabetic donor. Briefly, splenocytes (1 × 10^7^ cells) from the three treated groups, but not Saline-fed NOD mice, were mixed with splenocytes (1 × 10^7^ cells) from newly diabetic NOD mice and given by intravenous injection (i.v.) into the tail veins of 6–8-week-old NOD/SCID mice. Mice receiving only 1 × 10^7^ diabetic splenocytes were used as a control. Recipient mice were monitored for the development of diabetes for 15 weeks. 

### 2.16. Statistical Analysis

Survival analyses with Kaplan-Meier estimates were used to evaluate the difference in diabetes incidence among the NOD mice groups. Results were otherwise analyzed by using *t*-tests or ANOVA for multiple comparisons. A *P* value of less than  .05 was considered to indicate statistical significance in all cases. 

## 3. Results

### 3.1. Construction and Identification of the Recombinant BmNPV CTB-Ins-GFP

**Figure 1:**
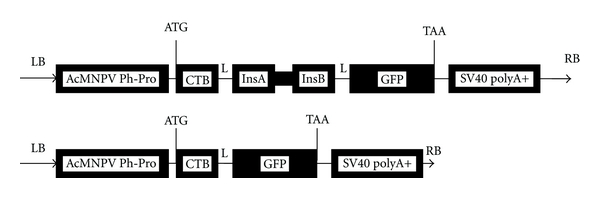
Schematic structure of the CTB-Ins-GFP fusion gene. AcMNPV Ph-Pro: *Autographa californica *multiple nuclear polyhedrosis virus polyhedrin promoter, CTB: cholera toxin B subunit, L: linker peptide (GPGP), InsA: human insulin subunit A, InsB: human insulin subunit B (InsA and InsB are linked by mini-C peptide (RRGSKR)), GFP: green fluorescent protein, and LB and RB: left and right border, respectively, of the donor vector pFastBac1.

Using pEGFP, PAK-CTB-INS, and five primers, we obtained the GFP, CTB, and CTB-Ins genes by traditional PCR. Using the SOE-PCR method, the GFP gene was anchored separately at the 3′-end of the CTB and CTB-Ins genes to form the CTB-GFP and CTB-Ins-GFP fusion genes, respectively. A flexible hinge tetrapeptide (GPGP) was introduced between the two peptides to maintain the natural conformation of CTB/CTB-Ins and GFP (Figure 1). The segments were confirmed by autosequencing and then subcloned into the pFastBac1 vector. Results of the enzyme digestion, PCR amplification, full-length nucleotide sequencing, and deduced amino acid sequences showed the fusion genes were exactly inserted into the pFastBac1 vector (data not shown). Then, they were transformed into the *E. coli* host strain DH10*α* (competent cells) to generate recombinant bacmid vectors. PCR results showed that the recombinant bacmid vectors were successfully constructed (data not shown). The BmN cells were transfected by the recombinant bacmid vectors to obtain recombinant viruses. Viral genomic DNA was extracted and recombined by PCR amplification (data not shown). According to the Reed-Muench formula, the dilutions of the recombinant viruses were 1.17 × 10^9^ for BmNPV CTB-Ins-GFP and 5.62 × 10^9^ for BmNPV CTB-GFP (data not shown). 

### 3.2. High Expression of the CTB-Ins-GFP Recombinant Protein

**Figure 2:**
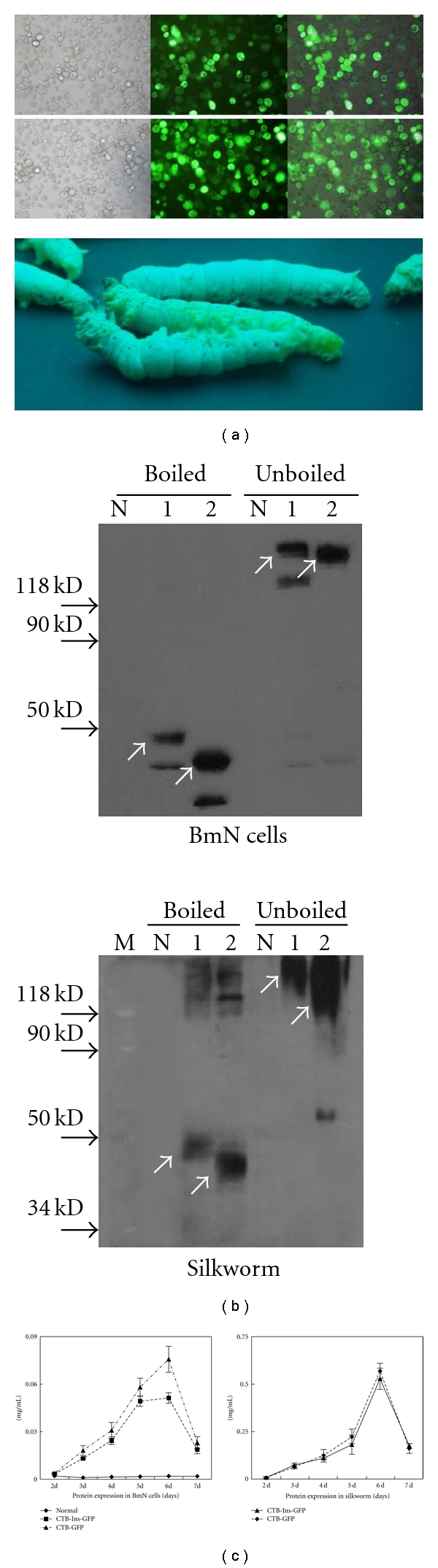
Identification of the expressed fusion proteins. (a) Fusion protein expression in BmN cells and silkworm *B. mori* fifth-instar larvae. Left to right: BmNPV CTB-GFP or BmNPV CTB-Ins-GFP 5 days after infection under normal light, blue light, and overlapped. Magnification, 200x. Lower: *B. mori* fifth-instar normal larvae and larvae infected with recombinant viruses 5 days after infection under ultraviolet radiation. (b) Western blot analysis of fusion protein expression. Lane N: control, lane 1: CTB-Ins-GFP protein, lane 2: CTB-GFP protein. GFP antibody was used as the primary antibody. Silkworm: lane M: molecular weight standard, lane N: normal sample, lane 1: CTB-Ins-GFP protein, lane 2: CTB-GFP protein. Anti-CTB serum was used as the primary antibody. The arrows denote the pentamers (~240, 195 kDa) and monomers (~48, 39 kDa) of the CTB-Ins-GFP and CTB-GFP proteins. (c) ELISA semiquantification analysis of protein expression. Concentrations on days after infection were calculated according to the standard curve of bacterial CTB. Data are presented as the mean concentration ± SD on each day. The experiment was repeated twice.

When infected with the recombinant virus, BmN cells and silkworm larvae showed virus infection symptoms and started to produce CTB-GFP and CTB-Ins-GFP fusion proteins in the cytoplasm and body cavity, respectively (Figure 2(a)). The presence of the proteins was determined by Western blot analysis with anti-CTB or -GFP antibodies (Figure 2(b)). We found that the oligomeric fusion proteins (CTB-GFP protein ~195 kDa; CTB-Ins-GFP protein ~240 kDa) were dissociated into monomers by boiling and subsequently migrated as a specific band with an apparent molecular weight (CTB-GFP protein ~39 kDa; CTB-Ins-GFP protein ~48 kDa), with normal samples as negative controls. No immunospecific signal corresponded in molecular mass to the CTB-GFP or CTB-Ins-GFP fusion proteins in normal cells or normal hemolymph samples. Moreover, the ELISA results showed that the highest detectable level of the CTB-GFP and CTB-Ins-GFP fusion proteins yielded by BmN cells was 0.037 mg/2 × 10^6^ cells on the 6th day after infection (Figure 2(c)). In the infected silkworm larvae, the recombinant fusion proteins were efficiently expressed and released into the larval hemolymph. On the 6th day after infection, the maximum amount of fusion protein in the hemolymph reached 0.58 mg/mL with an average of 0.4 mL hemolymph per larva (Figure 2(c)). The results demonstrated that the silkworm is a powerful “bioreactor” for protein production. 

### 3.3. Strong Affinity of the Recombinant Protein for the GM1 Ganglioside

**Figure 3:**
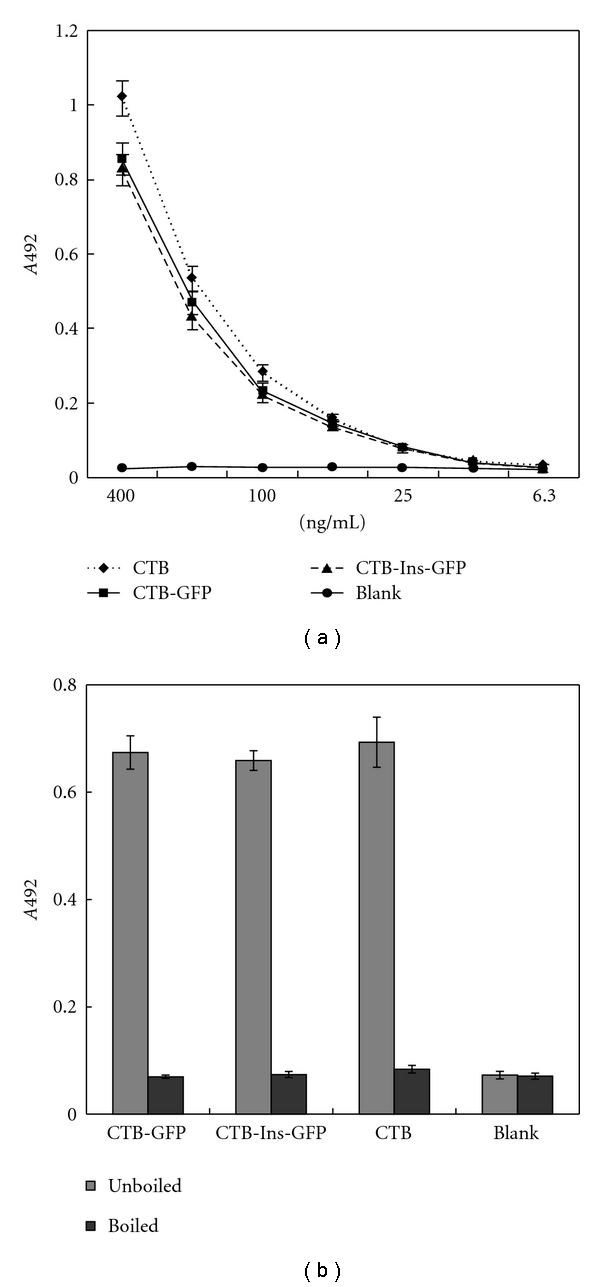
GM1 affinity assay. (a) Reactivity of the CTB-Ins-GFP protein with the GM1 ganglioside and a native bacterial CTB control. (b) Boiling induced pentamer dissociation into monomers. Approximately equal amounts of the three different samples indicated were used to measure A492 signal levels. Data represent the mean A492 values ± SD of each sample. The experiment was repeated twice.

The CTB-based protein in its natural conformation forms a pentameric structure and possesses an affinity for the GM1 ganglioside, so we used the GM1-ELISA method with GM1 ganglioside as the capture molecule to confirm the specific affinity of the fusion protein. An increase in the concentration-specific absorption signal was observed in contrast to the bacterial pentameric CTB standard curve, indicating that the recombinant protein existed as pentamers, because only pentameric protein binds to GM1 ganglioside (Figure 3(a)). However, the heat-treated protein lost its affinity for GM1 ganglioside (Figure 3(b)). The silkworm-derived recombinant protein exhibited the biochemical and antigenic properties of CTB necessary for the next study. The GM1-binding ability also suggested proper folding of the CTB-GFP and CTB-Ins-GFP molecules, resulting in a functional pentameric structure. 

### 3.4. Feeding of CTB-Ins-GFP Protein Reduces Insulitis and Suppresses Diabetes

**Figure 4:**
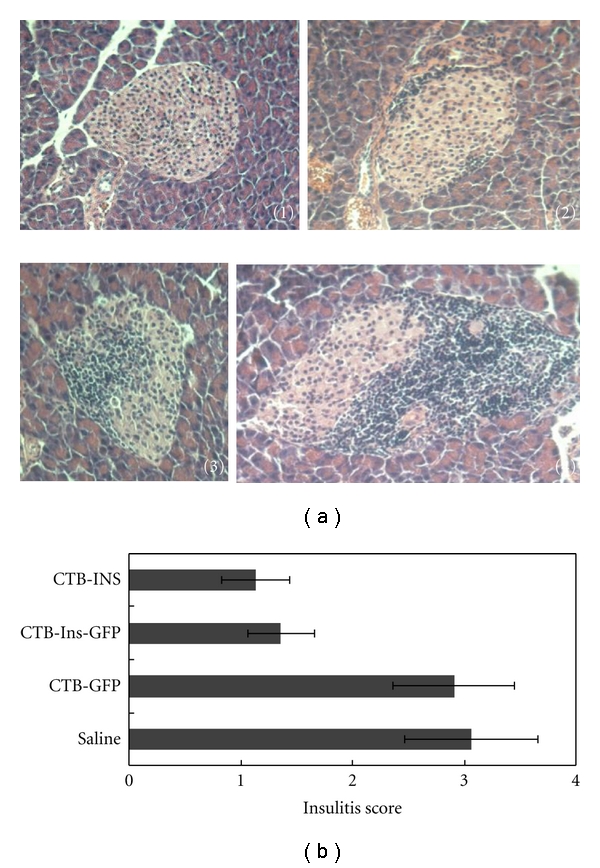
Insulitis score analysis. (a) Representative histopathological pancreatic islets from normal mice or experimental NOD mice. (b) Semiquantitative analysis of pancreatic islet insulitis score. Data are expressed as the mean score for each group ± SD. The ANOVA test revealed a significant reduction in insulitis in mice fed CTB-INS or CTB-Ins-GFP in contrast to the CTB-GFP or Saline group (1.1 ± 0.3 and 1.4 ± 0.3 versus 2.9 ± 0.5 and 3.1 ± 0.6, resp., *P* < .05). No significant difference in insulitis score was found in mice fed either CTB-GFP or Saline. Six mice per group were tested in two separate experiments.

To investigate the impact on insulitis as a result of feeding CTB-Ins-GFP protein to NOD mice, 5-week-old female NOD mice were given larval hemolymph containing recombinant CTB-GFP or CTB-Ins-GFP proteins or Saline, with a group fed hemolymph containing CTB-INS as a positive control, until 10 weeks. Then, their pancreatic tissues were collected and the islets individually scored for insulitis. Representative pancreatic islets from animals fed CTB-Ins-GFP or CTB-INS fusion proteins had insulitis scores of 1 and 2 (Figure 4(a)(2), (3)), whereas animals fed CTB-GFP or Saline had insulitis scores of 3 and 4 (Figure 4(a)(4)) with normal mice as a negative control (Figure 4(a)(1)). The ANOVA test revealed a significant reduction of insulitis in mice fed CTB-Ins-GFP or CTB-INS in contrast to the CTB-GFP or Saline groups (1.1 ± 0.3 and 1.4 ± 0.3 versus 2.9 ± 0.5 and 3.1 ± 0.6, resp.; *P* < .05; Figure 4(b)). No significant difference in insulitis scores was found in mice fed CTB-GFP or Saline. These results showed the CTB-Ins-GFP protein synthesized by the silkworm effectively suppressed insulitis.

**Figure 5:**
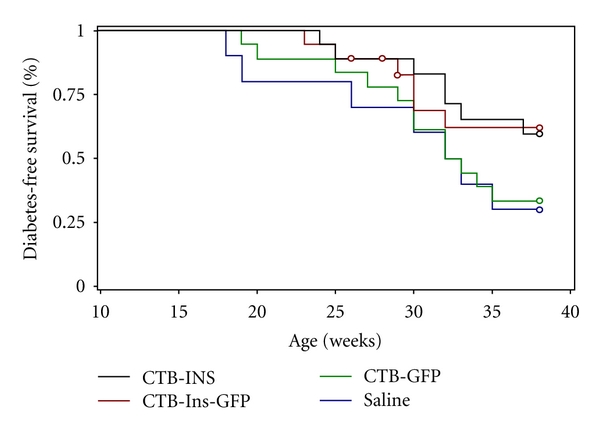
Effect of fusion protein feeding on diabetes development. Five-week-old female NOD mice were fed CTB-INS (group 1, *n* = 18, black), CTB-Ins-GFP (group 2, *n* = 18, red), CTB-GFP (group 3, *n* = 18, green), or Saline (group 4, *n* = 10, blue) four times per week until 38 weeks of age. Diabetes was confirmed by hyperglycemia (>16.7 mM glucose) for 2 consecutive weeks. The ANOVA test revealed a significant reduction in T1D onset in groups 1 and 2 compared with groups 3 and 4 (*P* = .0276). No significant difference was found between group 1 and group 2 (*P* = .9683).

We then investigated whether long-term oral administration of the CTB-Ins-GFP fusion protein could prevent diabetes. Prediabetic female NOD mice were orally administered the CTB-Ins-GFP fusion protein from 5 to 35 weeks of age with a CTB-INS protein-fed group as a control. In the early stages, mice in all groups had normal appetites and weight gains, and no apparent difference was observed (data not shown). Then, the mice were found to have a decrease in body weight and an increase in appetite (data not shown). At 35 weeks of age, only 39% and 33% (7/18 and 6/18) of the NOD mice that received CTB-INS (group 1) or CTB-Ins-GFP (group 2) fusion protein developed diabetes compared with 67% and 70% (12/18 and 7/10) of those fed the CTB-GFP peptide (group 3) or Saline (group 4), respectively (groups 1 and 2 versus groups 3 and 4, *P* = .0276; group 1 versus group 2, *P* = .9683; Figure 5). Although this result showed that feeding CTB-Ins-GFP fusion protein might not totally suppress diabetes development in NOD mice, the incidence of diabetes decreased markedly in the CTB-Ins-GFP- and CTB-INS protein-fed groups, compared with the CTB-GFP and Saline groups. The percentage of mice fed CTB-GFP alone did not differ significantly from the Saline control group. These results suggest that the CTB-Ins-GFP fusion protein, orally administered, may offer an alternative approach to effectively prevent or even treat diabetes. 

### 3.5. Interaction between the Recombinant Protein and Gut Mucosa

**Figure 6:**
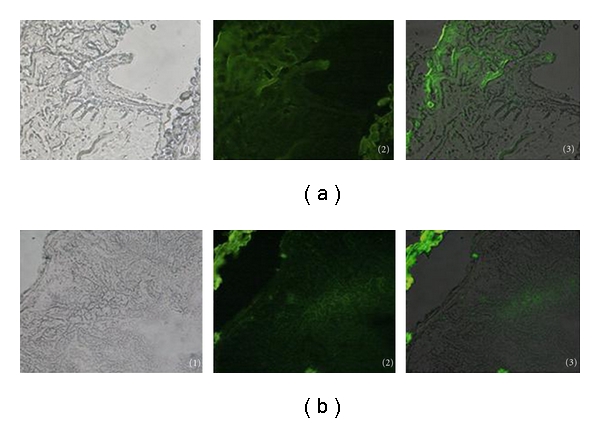
Intestinal mucosal binding assay. Images of the NOD mice frozen gut sections of (a)NOD mice fed CTB-GFP and (b) NOD mice fed CTB-Ins-GFP. Gut sections under (1) normal light, (2) blue light, and (3) overlapped. Magnification is 40x.

To verify the interaction of the fusion protein with the intestine, we obtained 8 *μ*m of frozen gut sections of NOD mice from the protein binding assay. Photomicrographs showed that the GFP tag was effectively located on the fusion proteins similarly to other tagged probes or antibodies, as expected (Figure 6). We concluded that the fusion GFP-tagged proteins had complete GFP ability and could be used to trace the process of protein transportation in the intestinal mucosa by direct visualization of the GFP tag. 

### 3.6. Feeding with CTB-Ins-GFP Fusion Protein Induced a Humoral Immune Response

**Figure 7:**
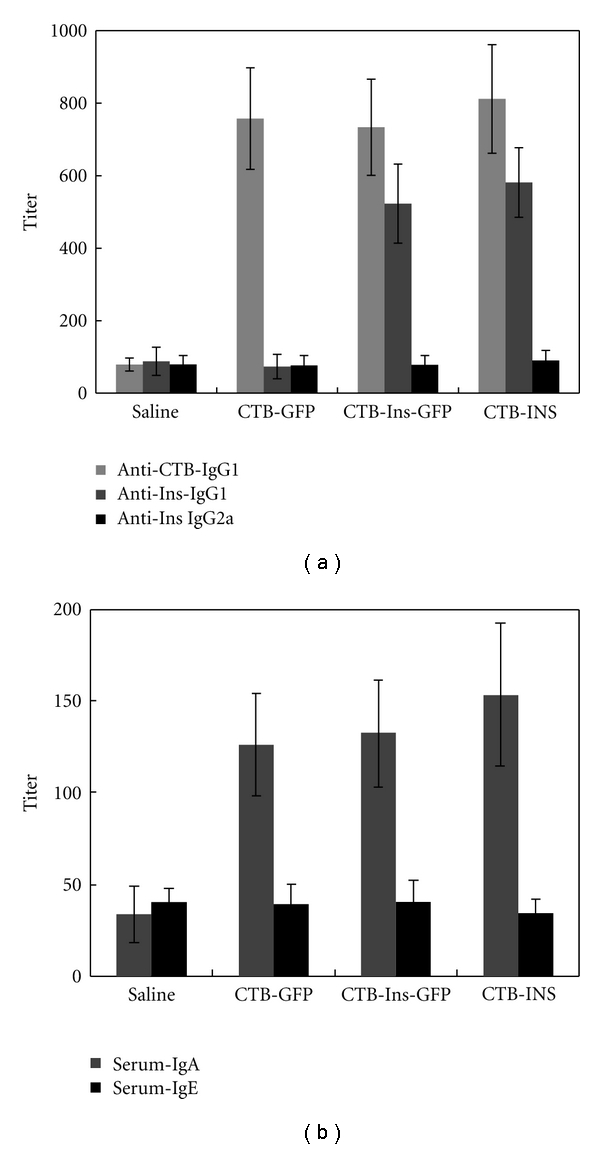
Serum antibody assay of treated NOD mice. (a) Anti-CTB IgG1, anti-insulin IgG1, and IgG2a serum antibody titers and (b) IgA and IgE serum titers in mice fed CTB-INS, CTB-Ins-GFP, CTB-GFP, or Saline. Results are presented as the mean titer values ± SD. Four or five mice per group were individually tested in two separate experiments.

Generally, oral administration of certain protein antigens can induce humoral immunoreactions to the antigen, so we assayed 10-week-old female NOD mouse anti-CTB and anti-insulin antibody serum titers to investigate whether feeding Saline (group 1), CTB-GFP (group 2), CTB-Ins-GFP (group 3), or CTB-INS (group 4) hemolymph would induce specific humoral immune responses. We found that NOD mice fed CTB-Ins-GFP or CTB-INS hemolymph had both anti-CTB and anti-insulin antibodies in their sera (data not shown). Serum anti-CTB IgG1 levels in animals fed CTB-GFP, CTB-Ins-GFP, or CTB-INS hemolymph were significantly higher than in the Saline group (antibody titer: 758 ± 140, 734 ± 133, and 812 ± 150 versus 79 ± 18, resp.; *n* = 9 per group; *P* < .05). A significant increase in serum IgG1 anti-insulin antibody titers in NOD mice fed CTB-Ins-GFP or CTB-INS hemolymph was observed compared with the CTB-GFP and Saline groups (antibody titer: 523 ± 109 and 581 ± 96 versus 73 ± 34 and 88 ± 39, resp.; *n* = 9 per group; *P* < .05), with no significant difference in serum IgG2a antibodies (Figure 7(a)). Additionally, IgA antibodies, rather than IgE, in the sera of groups 2–4 were elevated in contrast to group 1 (antibody titer: 126 ± 28, 133 ± 29, and 154 ± 39 versus 20 ± 6, resp.; *n* = 9 per group; *P* < .05; Figure 7(b)). These findings indicate that oral insulin-induced Th2 lymphocyte-mediated oral tolerance resulted in predominantly IgG1 rather than other antibody isotypes. 

### 3.7. Upregulation of Treg Cells in the Spleen, Intestinal Lymph Nodes, and Blood

We assayed the CD4^+^CD25^+^Foxp3^+^ T cells in the peripheral lymph tissues of 10-week-old female NOD mice by FACScan flow cytometry. The results showed that the proportions in CTB-INS and CTB-Ins-GFP fed NOD mice were significantly elevated in contrast to the CTB-GFP and Saline groups (2.87 ± 0.35% and 2.75 ± 0.34% versus 1.54 ± 0.42% and 1.45 ± 0.38%, resp., in the spleen, 3.16 ± 0.53% and 2.9 ± 0.42% versus 1.93 ± 0.29% and 1.82 ± 0.36%, resp., in intestinal lymph nodes, and 3.71 ± 0.78% and 3.61 ± 0.7% versus 2.19 ± 0.27% and 2.22 ± 0.14%, resp., in blood, *P* < .05; Table 2), which were correlated with the suppression of T1D onset, and all were higher than in normal mice (0.89 ± 0.11%, 1.01 ± 0.28%, and 1.5 ± 0.36% in the spleen, intestinal lymph nodes, and blood, resp.). However, in diabetic NOD mice, the CD4^+^CD25^+^Foxp3^+^ T cell proportions were much higher than that in any other group (5.42 ± 0.83%, 8.11 ± 1.6%, 20.63 ± 5.79% in the spleen, intestinal lymph nodes, and blood, resp., *P* < .05), and we believe that this was a result of a T1D autoimmune feedback mechanism. The results indicated that the induced oral tolerance specifically increased the proportions of CD4^+^CD25^+^Foxp3^+^ T cells, which might suppress T1D progression in the peripheral lymph system. 

### 3.8. Effect on Spleen Lymphocytes

**Figure 8:**
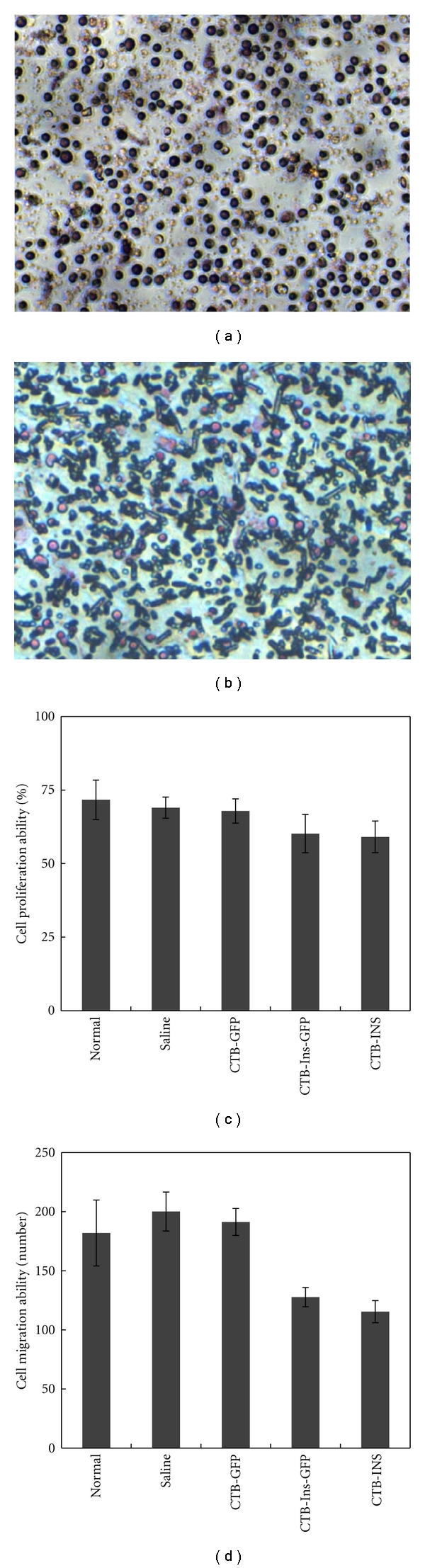
Proliferation and migration abilities of splenocytes in treated NOD mice. Splenocytes (1 × 10^7^ cells) isolated from CTB-INS-, CTB-Ins-GFP-, CTB-GFP-, Saline-fed and normal mice at 10 weeks of age were cultured in 24-well plates with 10 *μ*M BrdU for 24 h. Cells were immunohistochemically stained and examined for stained cell proportions (representative of proliferative ability) according to the protocol. Or splenocytes were cultured in the upper chamber of Transwell plates with 0.6 mL of normal culture medium (IL2 150 IU/mL) placed in the bottom chamber at 37°C with 5% CO_2_ for 14 h. The migrated cells were fixed, stained, and numbered under the microscope. A. Splenocyte BrdU assay. Magnification is 400x. (b) Splenocyte Transwell assay. The magnification is 400x. (c) Statistical analysis of proliferative ability. (d) Statistical analysis of migratory ability. All results are presented as the mean titer values ± SD. Five mice per group were tested in two separate experiments.

To demonstrate the putative suppressive regulatory effect of induced Treg (iTreg) cells, we determined the proliferative and migratory abilities of spleen cells in treated NOD mice *in vitro* by BrdU label analysis and a Transwell assay. The performances indicated that specific immune suppression existed in the CTB-INS- and CTB-Ins-GFP- treated NOD mice, manifested by the suppression of the ability of the cells to proliferate and migrate (proliferative ability: CTB-INS- and CTB-Ins-GFP-treated groups: 60.19 ± 6.51% and 59.1 ± 5.38%, resp., *P* = .00074, versus CTB-GFP and Saline treated groups or normal mice 67.87 ± 4.13%, 69.11 ± 3.6%, and 71.67 ± 6.73%, resp., *P* = .00009; migratory ability: CTB-INS and CTB-Ins-GFP- treated groups: 128 ± 8 and 115 ± 9, resp., *P* = .025, versus CTB-GFP- and Saline-treated groups or normal mice: 200 ± 17, 191 ± 11, and 182 ± 28, resp., *P* = .0068; Figure 8). This suggests that oral tolerance might suppress spleen lymphocyte activity via the iTreg cells in some way .

### 3.9. Adoptive Transfer of Treatment Regulatory Cells

**Figure 9:**
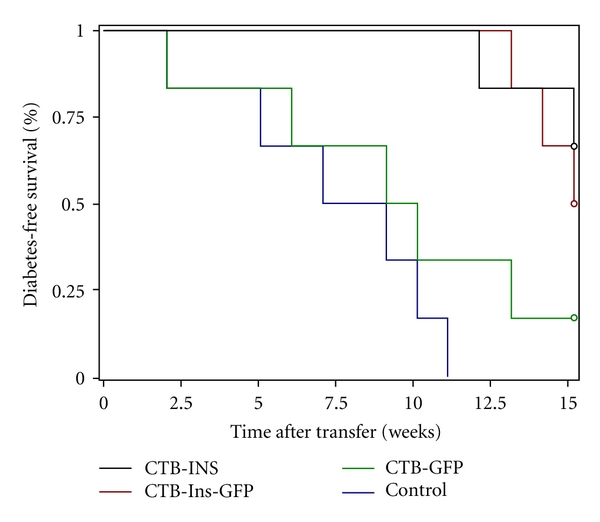
Splenocytes from insulin-fed NOD mice prevented diabetes after adoptive transfer. Splenocytes (1 × 10^7^ cells) isolated from CTB-INS- (group 1, black), CTB-Ins-GFP- (group 2, red), and CTB-GFP- (group 3, green) fed mice at 10 weeks of age were mixed with diabetogenic splenocytes (1 × 10^7^ cells) from diabetic NOD mice, and then cotransferred to 8-week-old NOD/SCID mice (*n* = 6 for all four groups) with a diabetogenic splenocyte-only injection (group 4, blue) as a control. The development of diabetes in the recipients was monitored for 15 weeks. The ANOVA test revealed a significant reduction in T1D onset in groups 1 and 2 compared with groups 3 and 4 (*P* = .0001). No significant difference was found between groups 3 and 4 (*P* = .6064).

To determine whether administration of the CTB-Ins-GFP protein protected NOD animals from the development of diabetes via some regulatory cells, we used an adoptive transfer model. Splenocytes from 10-week-old CTB-INS- (group 1), CTB-Ins-GFP- (group 2), and CTB-GFP (group 3)-fed NOD mice were mixed with diabetogenic splenocytes and given i.v. to 6–8-week-old syngeneic NOD/SCID recipient mice. Mice receiving only diabetogenic splenocytes (group 4) were used as a negative control. We found 83% and 100% (5/6 and 6/6) of the NOD/SCID recipient mice receiving splenocytes from group 3 and group 4 NOD mice, respectively, developed diabetes, whereas splenocytes from group 1 and group 2 NOD mice were able to prevent diabetes in 67% and 50% (4/6 and 3/6) of NOD/SCID mice, respectively (groups 1 and 2 versus groups 3 and 4, *P* = .0001; group 3 versus group 4, *P* = .6064; Figure 9). These results demonstrated that oral administration of CTB-INS or CTB-Ins-GFP fusion proteins generated some regulatory cells, including increased CD4^+^CD25^+^Foxp3^+^ Treg cells, which could transfer suppressive ability in NOD mice. 

## 4. Discussion

Oral tolerance is a potential therapeutic strategy, because it can induce “inappropriate” immune responses to specific “self” antigen proteins that precede overt autoimmune disease, such as T1D [1]. Human insulin is the major autoantigen orally administered in treating T1D via induction of special mucosal tolerance [6, 11, 19]. However, the obvious effect and application of this approach depends not only on the availability of autoantigens produced in quantity, but also on the detailed mechanisms of tolerance to make it amenable to clinical testing.

Repeated oral administration of large doses of insulin is essential in maintaining specific unresponsiveness for long periods because of the large absorptive intestinal mucosal area. The CTB peptide as a mucosal carrier was applied successfully to induce peripheral immunological tolerance [7, 11]. In previous studies, it was shown that specific tolerance could be enhanced by conjugating insulin to the CTB peptide [19]. The CTB-conjugated protein functions in a pentameric structure, interacting with GM1 ganglioside expressed on the intestinal epithelial cells, facilitating the mucosal immune reaction and increasing the autoantigen concentration. Others have reported that CTB may enhance the immune reaction by affecting the intestinal tight junction [24]. Moreover, Terahara et al. [13] found that orally administered CT induced villous M-like cells in mice. Comprehensive gene expression revealed that they share traits with both intestinal epithelial cells and PP M-cells. The latter play significant roles in mucosal immunity. In a previous study of T1D treatment in NOD mice by oral insulin, Zhang et al. [6] found that administration of 1 mg of porcine insulin was an effective oral dose. However, we were able to decrease the effective dose of the autoantigen in the active CTB pentameric form to about 50 *μ*g. Together, our data and the results from other researchers support the theory that low doses administrated for oral tolerance favor active suppression, whereas high doses favor clonal anergy/deletion [25, 26].

So far, CTB-based fusion proteins have been successfully achieved in *E. coli* [8], potatoes [27], tomatoes [28], tobacco [9], and even rice [10]. In this study, we used silkworm to express the fusion proteins by insect BES. The insect BES was first used in 1993 to obtain the cytokine interferon-*α* and has been broadly applied to produce proteins due to its numerous virtues [20]. In recent years, the improved BES, the so-called Bac-to-Bac BES, has been used more, because it is quicker, more efficient, and allows easier identification with lower workloads [29]. In the present study, we produced protein at levels up to 0.58 mg/mL. Additionally, the proteinase inhibitors [30] and biocapsule-like fat of silkworms may keep the protein of interest stable and protect it against gastrointestinal degradation, making the hemolymph especially appropriate for oral administration. The results indicate that Bac-to-Bac BES is suitable for producing the large quantities of proteins or polypeptides of interest.

The precise mechanism by which the CTB-Ins-GFP fusion protein enhances oral tolerance remains unclear. As a popular fluorescent protein first discovered in 1962, GFP was expressed in *E. coli* to reveal that FimH+ bacteria combine glycoprotein 2 of the intestinal M-cells to initiate a mucosal immune response [14]. It was also useful in work on the localization, structure, and dynamic processes of molecules, cells, and organisms, such as viruses, and even in interaction studies among DNA, RNA, and proteins [31, 32]. In the present study, the fusion protein was shown to interact with the gut mucosa, but appeared in neither the spleen nor the intestinal lymph nodes (data not shown). The orally administered protein has a low bioavailability, less than 3.1% [33]. It was previously reported that low amounts of insulin induced regulatory CD4^+^ T-cells [34–36]. In other diseases, like the arthritis mouse model, orally administered type II collagen was shown to induce CD4^+^CD25^+^Foxp3^+^ regulatory cells by IDO expressing CD11c^+^ DCs in intestinal Peyer's patches in the treatment of arthritis [37–39]. Moreover, oral tolerance induced by OVA coupled to CTB promoting OVA specific regulatory CD4^+^CD25^+^Foxp3^+^ T-cells was dependent on B lymphocytes not only in the mucosal inductive draining lymph node but also at the inflamed effector sites to suppress effector T-cells [16, 40, 41]. It was also observed that the expression of CD86 in B-cells decreased, whereas IL-10 producing CD19^+^ B-cells simultaneously increased. These Treg cells were reported to induce other CD4^+^ cells into becoming suppressors in addition to inducing the apoptosis or depletion of effector T-cells by contact-dependent or cytokine-dependent action mechanisms. According with this, our results indicate that CTB enhances immunomodulation of insulin by the humoral and Th2-like immune responses and induction of CD4^+^CD25^+^Foxp3^+^ regulatory T cells. The adoptive cotransfer experiment also showed that the regulatory cells were functionally active. This is probably because the autoantigen insulin plays an important role in T cell differentiation into Treg cell lineages. Administration of exogenous CTB-Ins-GFP fusion protein may create a special microenvironment in the gut that can promote beneficial humoral Th2-like immune responses, instead of harmful Th1-like immune reactions [42–44], and also insulin-specific regulatory T cells by some immature DCs or special B cells in the intestine, which play an essential role in the development of spleen cells [45]. Although the exact process by which the protein treats T1D remains to be discovered, these findings validate the use of the autoantigen insulin as an ideal immunotherapy strategy in the treatment of diseases involving CD4^+^CD25^+^Foxp3^+^ Treg cells.

Treg cells are attracting great interest because of their potential therapeutic role in autoimmune diseases [46]. To some extent, T1D can be “cured” by pancreatic transplantation [47], gene therapy [48, 49], or injection of specific autoantibodies, such as anti-CD3 antibodies [50]. However, none of these meet the requirements of an ideal cure [51]. Our work offers support for the use of oral tolerance in treating T1D. Generally, our data and other studies suggest that the antigen amount, delivery mode, and/or coadministered adjuvant molecules may have profound effects on directing the mechanism of tolerance. Successful application of oral tolerance in the treatment of human diseases will depend not only on the detailed functional mechanisms but also on strategies that can improve antigen presentation, alter the administered dose and formulations, use potent mucosal adjuvants, target the correct cells in the gut-liver axis, develop immune biomarkers for detecting effects, use combination therapies with other immunomodulatory agents, and target the right patient populations [52–55]. 

## 5. Conclusions

In summary, this study demonstrates that low doses of orally administered silkworm-produced CTB-Ins-GFP protein can effectively suppress the development of diabetes in NOD mice, where the treatment function involves inducing CD4^+^CD25^+^Foxp3^+^ Treg cells. Our experimental evidence lends further support to the hypothesis that orally administering the CTB-Ins protein may be a feasible way to treat T1D patients, and oral tolerance may be a powerful therapeutic strategy for treating autoimmune diseases. 

## Figures and Tables

**Table 1:** Five primers synthesized for the construction of the fusion proteins.

No.	Sequence	Length
P1	5′-CG*GGATCC*ATGATTAAATTAAAATTTGG-3′ (BamHI)	28 bp
P2	5′-**GGGGCCGGGGCC**GTTGCAGTAGTTCTCCA-3′ (GPGP)	29 bp
P3	5′-**GGGGCCGGGGCC**ATTTGCCATACTAAT-3′ (GPGP)	27 bp
P4	5′-**GGCCCCCGGCCCC**GTGAGCAAGGGCGAGGA-3′ (GPGP)	29 bp
P5	5′-TG*CTCGAG*TTACTTGTACAGCTCGTCC-3′ (XhoI)	27 bp

The italics in P1 and P5 indicate BamHI and XhoI restriction endonuclease sites, respectively. Bold type indicates the linker peptide (GPGP).

**Table 2:** Treg cell proportion in the peripheral lymph tissues of NOD mice.

Type of mice	Material oral administrated	CD4^+^CD25^+^Foxp3^+^ T cell's proportion (%)					
		Nondiabetes			Diabetes		
		Spleen	Lymph node	Blood	Spleen	Lymph node	Blood
NOD mice	Saline	1.45 ± 0.38	1.82 ± 0.36	2.22 ± 0.14	5.42 ± 0.83	8.11 ± 1.6	20.63 ± 5.79
	CTB-GFP	1.54 ± 0.42	1.93 ± 0.29	2.19 ± 0.2			
	CTB-Ins-GFP	2.75 ± 0.34*	2.9 ± 0.42*	3.61 ± 0.70*			
	CTB-INS	2.87 ± 0.35****	3.16 ± 0.53*	3.71 ± 0.78*			

Normal mice		0.89 ± 0.11	± 0.28	1.5 ± 0.36			

Splenocyte (1 × 10^5^ cells) isolated from CTB-INS, CTB-Ins-GFP, CTB-GFP, Saline-fed NOD mice at 10 weeks of age were treated with the mouse regulatory T cell staining kit by the protocol. Splenocytes of the normal mice and the diabetic NOD mice (from the diabetes onset experiment) was used as contrasts. These treated splenocytes were examined by FACScan flow cytometer for CD4^+^CD25^+^Foxp3^+^ Treg cell proportions. Results are presented as the mean titer values ± S.D. Four mice per group were tested in two separate experiments.

*Values *P* < .05 is for statistical differences among mice groups treated with CTB-INS, CTB-Ins-GFP, CTB-GFP, and Saline.
